# Extension of the Moore–Greitzer framework for accurate mathematical modelling of flow instabilities in a low-speed isolated axial compressor rotor blades row

**DOI:** 10.1038/s41598-026-50460-y

**Published:** 2026-05-01

**Authors:** Marzieh Katebi, Reza Taghavi Zenouz

**Affiliations:** Aerodynamic and Turbomachine Research Laboratory, School of Mechanical Engineering, Iran University of Science and Technology (IUST), Narmak Tehran, 16846-13114 Iran

**Keywords:** Axial compressor, Flow instabilities, Surge, Rotating stall, Moore-Greitzer model, Engineering, Mathematics and computing

## Abstract

The mathematical models presented for describing flow instabilities in compressors rely on parameters that are not directly related to physical characteristics of machines, which may lead to inaccurate results. The most frequent model used for predicting flow instabilities in axial compressors is the Moore-Greitzer model; however, its predictive accuracy is strongly affected by the simplified representation of the performance characteristic. The present study offers modifications to this method through utilizing experimental performance curves and reconstructing it using a piecewise cubic polynomial formulation. Analysis of the B-Greitzer parameter for low-speed isolated axial compressor rotor blades row identifies a critical threshold at B = 0.71 marking transition between rotating stall and surge. As the B parameter increases, both the frequency and amplitude of flow oscillations are affected. The predictive capability of the original and modified models is assessed against experimental data. The proposed method significantly improves accuracy, reducing RMS errors from 11.97 to 0.89% for loading factor and from 5.65 to 0.81% for flow coefficient, providing a reliable and practical framework for real-world applications. Alongside ensured safety operation, this refined model has the potential of implementation in advanced control strategies, enabling compressors to operate more efficiently near the instability boundaries.

## Introduction

Compressors play a fundamental role in various industrial and aerospace applications. However, their performance and efficiency are frequently constrained by onset of flow instabilities. In general, two types of aerodynamic instabilities, known as rotating stall and surge, commonly affect the stability of both the axial and centrifugal compressors^1^. Surge is a system-wide instability characterized by large amplitude oscillations in the annulus flow throughout the entire compression system. In contrast, rotating stall is a more localized disturbance, potentially confined to specific stages of the compressor^2^.

The issue of instability, marked by phenomena such as stall and surge, has posed a significant engineering challenge since the early development of gas turbines. This problem remains critical due to its substantial impact on the stability and lifespan of compressor systems in gas turbine engines^3^. Degradation of stability in axial compressors at high pressure ratios is still a significant limitation for operation at higher efficiencies. Furthermore, the occurrence of these phenomena can lead to catastrophic system failure due to excessive mechanical forces or thermal loads on the blades.

So far, substantial research efforts have been directed towards the development of accurate mathematical models to describe the behaviors associated with surge and rotating stall. As summarized in Table 1, various theoretical and semi-empirical models have been proposed to better understand and predict the dynamics of surge and rotating stall^4–23^.

**Table 1:** Some of most notable mathematical models in predicting of flow instabilities in dynamic compressors.

References	Year	Flow description	Model description	Compressor type	Instability type
Greitzer^4,5^	1976	1D Incompressible	–	Axial	Surge
Hansen et al.^6^	1981	1D Incompressible	–	Centrifugal	Surge
Macdougal and Edler^7^	1983	1D Compressible	–	Axial/Centrifugal	Surge
Edler and Gill^8^	1985	1D Compressible	–	Centrifugal	Surge
Fink et al.^11^	1992	1D Incompressible	Including speed variations	Centrifugal	Surge
Botros^12^	1994	1D Compressible	Including speed variation	Axial/Centrifugal	Surge
Badmus et al.^13^	1995	Quasi-1D Compressible	–	Axial/Centrifugal	Surge
Gravdahl and Egeland^14^	1999	1D Incompressible	Including speed variations	Centrifugal	Surge
Moore and Greitzer^15,16^	1986	2D Incompressible	–	Axial	Surge/Rotating Stall
Feulner et al.^17^	1996	1D/2D Compressible	–	Axial	Surge/Rotating Stall
Ishii and Kashiwabara^18^	1996	2D Compressible	–	Axial	Surge/Rotating Stall
Gravdahl and Egeland^19^	1999	2D Incompressible	Including speed variations	Axial	Surge/Rotating Stall
Willms^20^	2000	1D Incompressible	–	Axial	Surge
Spakovszky I^21^	2002	2D Incompressible	–	Axial	Surge/Rotating Stall
Spakovszky II^22^	2003	2D Incompressible	–	Axial	Spatial harmonics
Shahriyari and Khaleghi^23^	2021	2D Incompressible	Including transient behavior	Axial	Surge/Rotating Stall

In 1976, Greitzer introduced a mathematical model of unstable behavior of compressing units^4^ and then, verified it empirically^5^. Moore developed a model of unsteady pressure rise across a blades passage at in-stall conditions^24^. The stall cell was modeled as a small circumferential disturbance to axial and tangential velocities. The disturbances were assumed to be expressed as Fourier series and the propagation velocity of the stall cell was calculated by equating the coefficients of trigonometric functions. Later, in 1986, a development towards the model, capable of capturing the shapes of fully formed transients, has been made by Moore and Greitzer^15,16^. Although their model has many simplistic assumptions, it has been commonly used for analytical investigations of the surge and rotating stall phenomena. The rotating stall inception may follow different routes commonly classified as modal (long-length scale) and spike-type (short-length scale) mechanisms. The Moore–Greitzer framework, is formulated as an annulus-averaged, lumped-parameter dynamic model, in which circumferentially averaged flow quantities describe the global dynamic response. By construction, this modeling structure captures large-scale dynamic behavior associated with modal stall development.

Despite its 38-year history, the Moore-Greitzer remains a subject of interest for researchers worldwide. It is regarded as a significant milestone and the foundation for the development of enhanced models tailored for specific applications. Shahriyari et al.^23^ developed a model based on Moore-Greitzer equations by adding the second-order derivative of the flow coefficient to the compressor pressure rise function. Shahriyari et al.^25^ compared the bifurcations and closed-loop performances of two compressor models,

Moore-Greitzer (MG) and a developed model based on MG (Shahriyari Khaleghi, SK), and demonstrating that while both models can stabilize small disturbances using LQR controllers, the SK model more accurately predicts instability under extreme perturbations, aligning closer with real industrial behavior. Meng et al.^26^ extended the Moore-Greitzer model to stochastic partial differential equations, allowing low-dimensional approximations that capture noise-induced surge and stall instabilities in axial-flow compressors, which provides a framework for more reliable and efficient jet engine design. However, the Moore-Greitzer model is based on parameters that are not directly related to the real characteristics of machines, and the selection of these parameters is not always obvious. Consequently, the precision of the calculations is contingent upon the accuracy of the parameters selected, which may lead to erroneous outcomes, including inaccurate surge and stall predictions.

Precise predictions of surge and rotating stall phenomena are critical for prevention of compressor failure. The currently operating strategy used in gas turbine engines is understood as “surge avoidance”, which is a passive approach and maintains stable operation at a sufficient margin from the surge line. In this strategy, the compressor cannot work near the surge line, where the pressure rise is maximum^27^.

If the initiation of the surge and rotating stall are precisely recognized, it would be possible to get closer to their critical boundaries, which could result in machine operation with at higher pressure ratio and efficiency. In this regard, the present study examined the applicability of the Moore-Greitzer model for a real machine. The method utilizes characteristics and experimental results of a low-speed axial compressor which has already been tested in a suitable test-rig of the Aerodynamic and Turbomachine Research Laboratory of the Iran University of Science and Technology (IUST-ATlab). Experimental results provided to declare that the process of the proposed compressor modeling is accurate. Relevant mathematical results were used to determine the value of the Moore and Greitzer’s parameter for the selected operational point. The employment of this method has enabled the successful implementation of the proposed model for reliable surge and stall protection.

## A review on Moore and Greitzer model

**Fig. 1:**
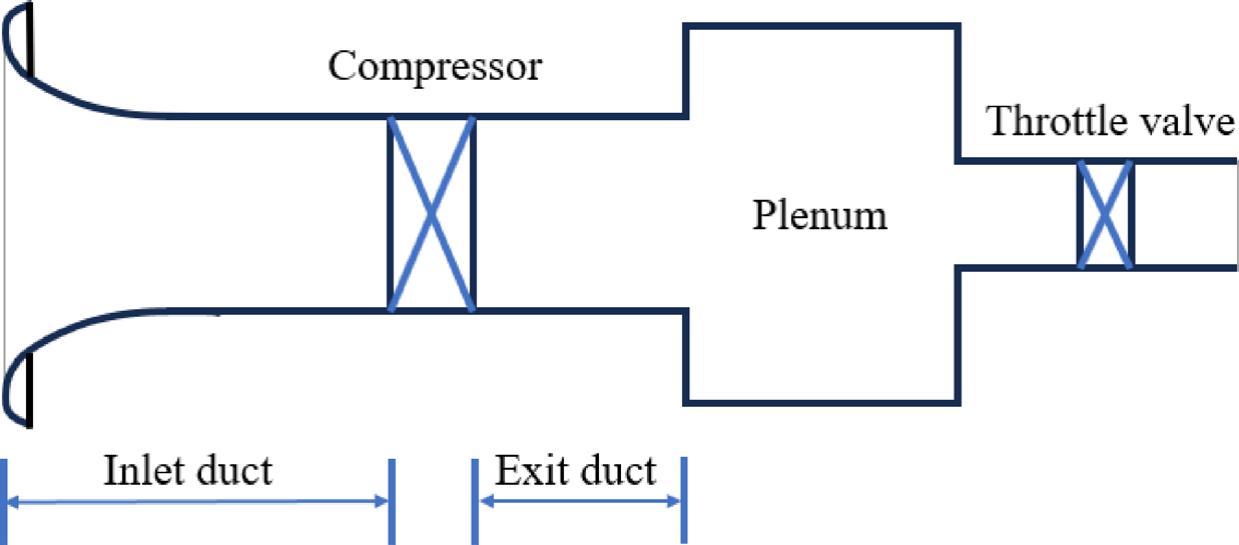
Compression scheme for mathematical modelling.

In 1986, Moore and Greitzer^15^ enhanced Greitzer’s compressor system-level model by incorporating a conceptual representation of the blade passage flows. This advancement resulted in a model capable of capturing both the rotating stall and surge phenomena in axial compressors, a capability that was experimentally validated in the same year^16^. This model is based on a compression scheme which is presented in Fig. 1 It consists of three parts: a compressor unit, a plenum and a throttle valve.

The compressing part of the system is modeled as a pipe with a constant cross-sectional area of A_c_ and a total length of L_c_. Compression is accomplished by a virtual piston generating the pressure rise. The throttling system is defined similarly, but without any piston. It is modeled as a pipe that can be closed at one end by reducing the outlet area. These pipes are connected to a plenum which is assumed to be a large reservoir in which the kinetic energy is neglected, but can be under various pressures denoted by p_p_. The volume of this plenum is designated as V_p_. In the context of a real machine, this plenum represents all the components and connections between the compressor unit and the throttling system.

The developed theory is based on the following assumptions:

- High root-to-tip ratio of blades row
- Non-rotational and inviscid flow in the inlet duct
- Axially symmetric disturbances in the plenum and gas duct
- Incompressible flow in all sections, except the plenum
- Negligible fluid velocity and acceleration in the plenum
- Uniform pressure distribution in the plenum at any instance
- Polytropic compression process
- Throttle valve of sufficiently short length. So that the flow acceleration in the throttle duct can be neglected.

In the Moore-Greitzer model, the momentum and continuity equations are used to describe the dynamic behavior of the compression system. Following, the procedures thoroughly explored in^15^, the general form of governing equations of the Moore-Greitzer model are introduced by Eqs. 1–3.

- Local circumferential momentum balance:
  1 $$\psi (\xi )+{l}_{c}\frac{d\phi (\xi )}{d\xi }={\Psi}_{c}\left(\Phi -{Y}_{\theta \theta }\right)-m{Y}_{\xi }+\frac{1}{2a}(2{Y}_{\xi \theta \theta }+{Y}_{\theta \theta \theta })$$
- Annulus averaged momentum balance:
  2 $$\psi (\xi )+{l}_{c}\frac{d\phi (\xi )}{d\xi }=\frac{1}{2\pi }{\int}_{0}^{2\pi }{\Psi}_{c}(\Phi -{Y}_{\theta \theta })d\theta$$
- Mass balance:
  3 $${l}_{c}\frac{d\psi (\xi )}{d\xi }=\frac{1}{4{B}^{2}}[\phi (\xi )-{\Phi}_{\mathrm{T}}]$$

All the quantities appeared in the above equations are specified in their non-dimensional form. The variables used in the above equations are as follows:

$\xi$ : Nondimensional time (Ut/R),

$\theta$ : Circumferential position around the annulus,

Y : Disturbance potential at the compressor entrance,

a : Reciprocal time lag parameter of blade passage,

${\psi}_{c}$ : Pressure rise coefficient of the axial compressor characteristic curve (axisymmetric pressure rise coefficient),

m: Outside compressor lag parameter.

a: Time lag parameter, and.

*lc*: Equivalent compressor length.

*li*: Inlet duct length.

*le*: Exit duct length.

A complete understanding of the equations of the Moore-Greitzer model requires also the definition of a few coefficients and parameters, as follows.

- Total-to-static pressure rise coefficient: $\psi =\frac{{P}_{s}-{P}_{T}}{\frac{1}{2}{\rho U}^{2}}$
- Annulus averaged flow coefficient: $\Phi ={\mathrm{C}}_{\mathrm{x}}/U$
- Axial flow coefficient: $\Phi \left(\xi \right)=\frac{1}{2\pi }{\int}_{0}^{2\pi }\phi (\theta ,\xi )d\theta$
- Throttle flow coefficient: ${\Phi}_{\mathrm{T}}=\left({{\mathrm{F}}_{\mathrm{T}}}^{-1}\left(\Psi \right)\right)=\gamma \sqrt{\Psi } , \mathrm{w}\mathrm{i}\mathrm{t}\mathrm{h} \gamma$ as the throttle gain
- Effective flow passage length through compressor and ducts: ${l}_{c}={l}_{i}+{\frac{1}{a}+l}_{e}$
- Greitzer parameter: $$B=\frac{{U}_{tip}}{2{L}_{c}{\omega}_{h}}$$

B parameter is in fact as a stability parameter, since its value determines if the system is stable or not. ${\omega}_{h} \mathrm{i}\mathrm{s} \mathrm{t}\mathrm{h}\mathrm{e}$ Helmholtz frequency.

Greitzer found out that there is a critical value of *B* (*Bcrit*) where the dynamic behavior of the compression system transitions from rotating stall to surge. For *B* > *Bcrit* the system exhibits large-amplitude oscillations in the mass flow and pressure which are associated with flow instability of the surge type. Conversely, for $B\le {B}_{crit}$, the instability manifests as rotating stall, characterized by reduced mass flow and pressure rise. Further details and process of deriving the governing equations of the Moore-Greitzer model are elaborated in^15^.

In the present study, the data of the test compressor is used to approximate the compressor characteristic curve and find the critical value of the *B* parameter. In addition, detailed information is obtained concerning compressor transient operation during the rotating stall and surge phenomena.

## Test model and experimental set-up

**Fig. 2:**
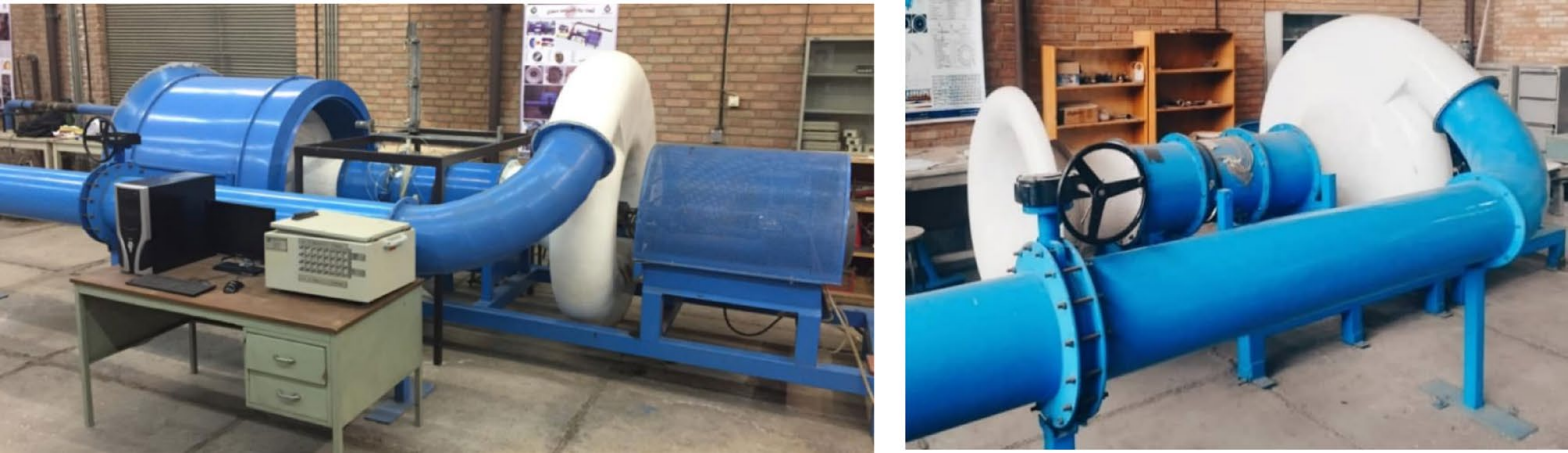
IUST-ATLab low-speed axial compressor test-rig.

**Fig. 3:**
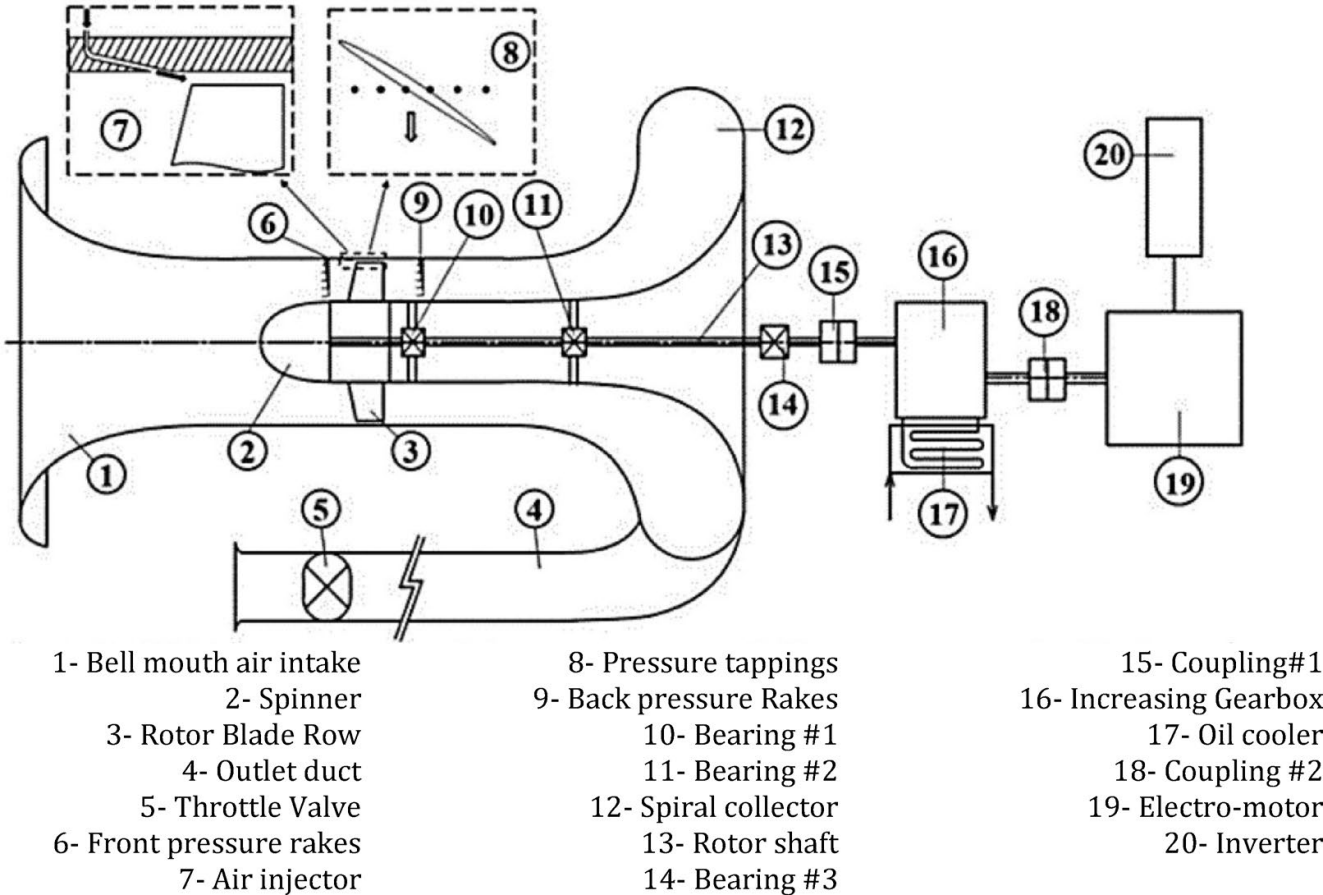
Layout of IUST-ACTLab low-speed axial compressor test-rig with its main components.

Experimental tests were carried out on an axial compressor single rotor blades row suitably mounted in a low-speed test-rig located at the Aerodynamic and Turbomachine Research Laboratory of the Iran University of Science and Technology (IUST-ATLab). A photograph and schematic drawing of this test-rig are shown in Figs. 2 and 3, respectively. Air stream enters the test-rig through a bell-mouth intake (item1) while running the compressor (item 3). Flow is being pressurized and then enters a spiral collector (item 12) and the following outlet duct (item 4). A throttle valve (item 5), located at the end of outlet duct, was used for adjusting the main mass flow rate. The position of the throttle valve was described by the dimensionless throttle opening parameter (TO). TO = 0% and 100% correspond to a fully closed and fully open valve, respectively. The compressor axis was driven by an electro-motor (item 19) which its speed was controlled via a frequency inverter (item 20).

**Fig. 4:**
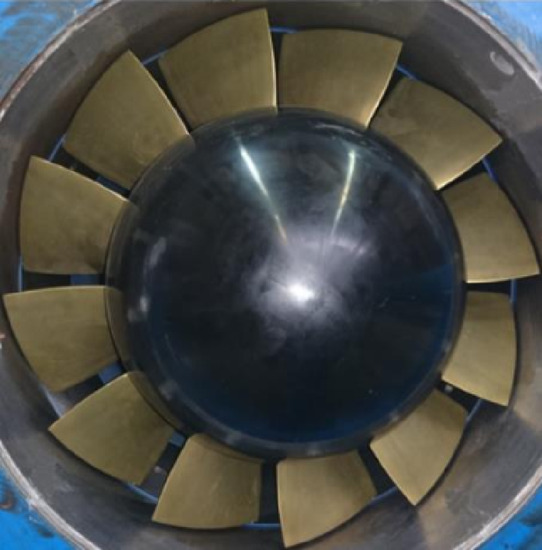
Rotor blades row of IUST-ATLab axial compressor.

The rotor blades row includes 12 blades with radial cross-sections based on the NACA-65 series airfoils. Figure 4 shows frontal view of this rotor blades row with a hub to tip ratio of 0.6. Its other geometrical specifications are presented in Table 2. This rotor has already been tested by Inoue^9^.

**Table 2:** Rotor blades row specifications.

Parameter	Unit	Hub	Mid	Tip
Radius	mm	135	180	224.5
Chord length	mm	106.1	117.8	117.5
Axial chord length	mm	90.46	80.03	65.36
Camber	mm	1.3	0.62	0.32
Solidity	–	1.5	1.25	1
Max thickness/chord	%	10	8	6
T.E. thickness/chord	%	1.41	1.27	1.28
Stagger angle	deg	31.5	47.2	56.2
Inlet blade angle	deg	49.1	57	62.5
Exit blade angle	deg	22.2	44.4	55.8

Taghavi et al.^10^ have already conducted many tests on this rotor blades row. A proper number of pressure tappings and hot-wire probes were used for logging the time averaged and instantaneous pressures and flow velocities in various locations. The pressure tappings and hot-wire probes were radially and circumferentially distributed at entry and exit regions of the rotor blades row.

## Compressor modeling

In compressor modeling process, a crucial step involves approximating the performance curves based on the data gained from various operational points. Under stable working conditions, i.e., the negative slope part of the performance curve, plotting these points is straightforward due to relatively stable pressure signals. However, the scenario changes significantly during rotating stall and surge events, where the pressure signals become highly unstable, i.e., over the positive slope part of the performance curve. This in turn, causes the modeling process to become more complicated.

**Fig. 5:**
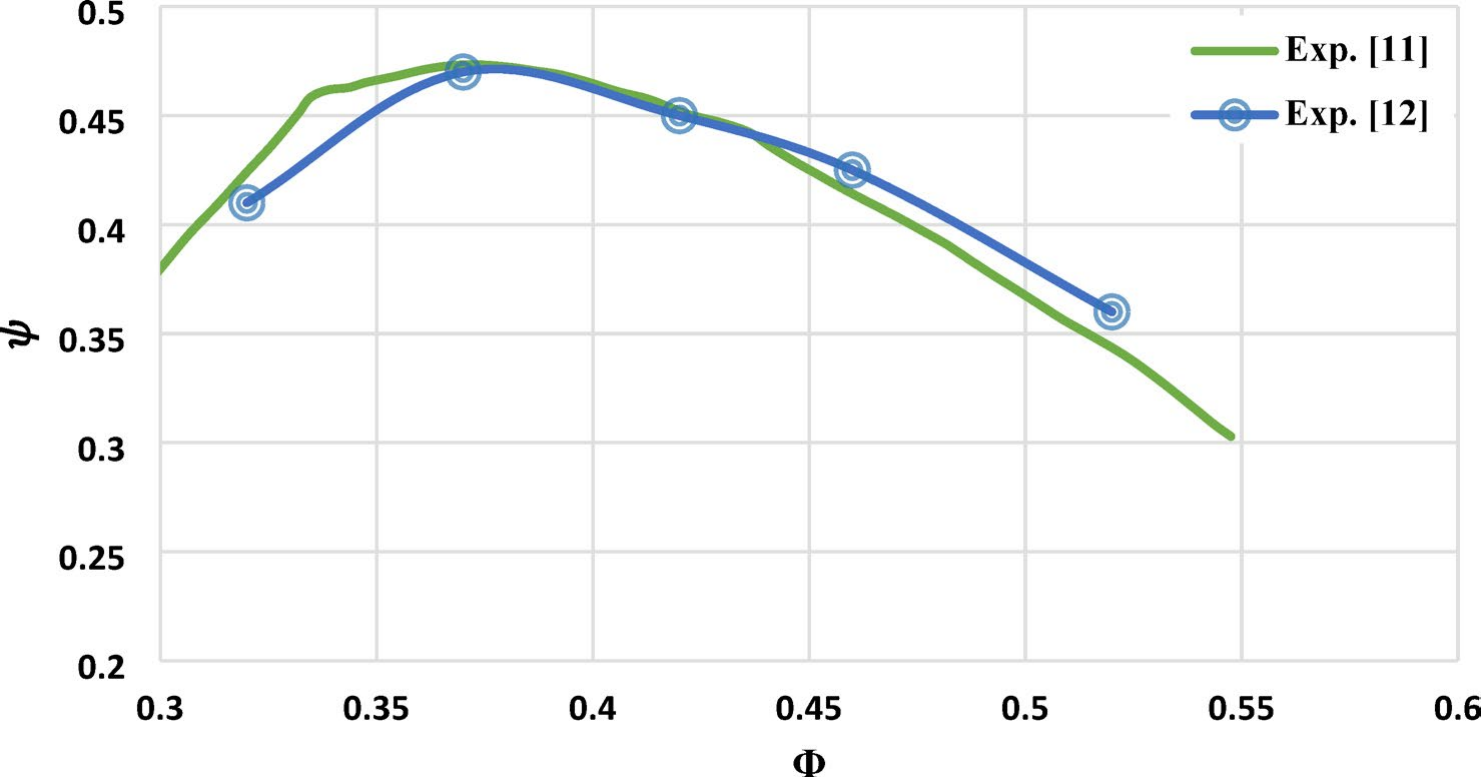
Experimental performance curve of the test axial compressor.

Figure 5 shows the performance curve of the test compressor obtained by Inoue^9^ and Taghavi et al.^10^, which shows very close agreement. These similar results have been used to mathematically approximate the compressor performance curve.

**Fig. 6:**
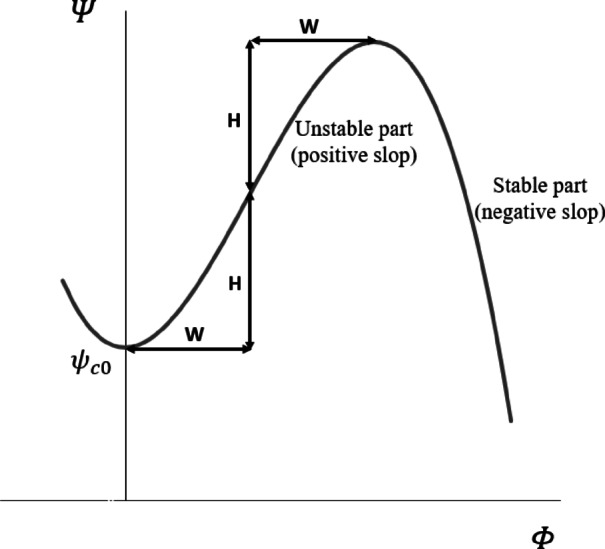
Generalized compressor performance map based on Moore-Greitzer model.

A significant contribution of the Moore-Greitzer model is its ability to describe the typical characteristics of the compressor map through a straightforward analytical expression. This is illustrated in Fig. 6, which exhibits a third-order polynomial curve representing the relationship between the flow coefficient and the pressure rise coefficient of the system, in accordance with Eq. 4.

4 $${\psi}_{c}\left(\phi \right)={\psi}_{c0}+H\left[1+\frac{3}{2}\left(\frac{\Phi }{\mathrm{W}}-1\right)-\frac{1}{2}{\left(\frac{\Phi }{W}-1\right)}^{3}\right]$$

The parameters appeared in the above equations are defined as follows:

${\psi}_{c0}$: compressor pressure rise coefficient at zero mass flow rate,

H: semi-height of cubic axisymmetric characteristic, and

$W$: semi-width of cubic characteristic.

The values of the aforementioned parameters will be obtained based on the experimental results.

The most important challenge is the inability of this method to precisely fit all experimental data points. The curves corresponding to the stable (negative slop) and unstable (positive slop) operations of the current compressor are described by Eqs. 5 and 6, respectively.

5 $${\psi}_{c}\left(\phi \right)=0.25+0.11\left[1+\frac{3}{2}\left(\frac{\Phi }{0.1863}-1\right)-\frac{1}{2}{\left(\frac{\Phi }{0.1863}-1\right)}^{3}\right]$$

6 $${\psi}_{c}\left(\phi \right)=-0.22+0.346\left[1+\frac{3}{2}\left(\frac{\Phi }{0.19}-1\right)-\frac{1}{2}{\left(\frac{\Phi }{0.19}-1\right)}^{3}\right]$$

**Fig. 7:**
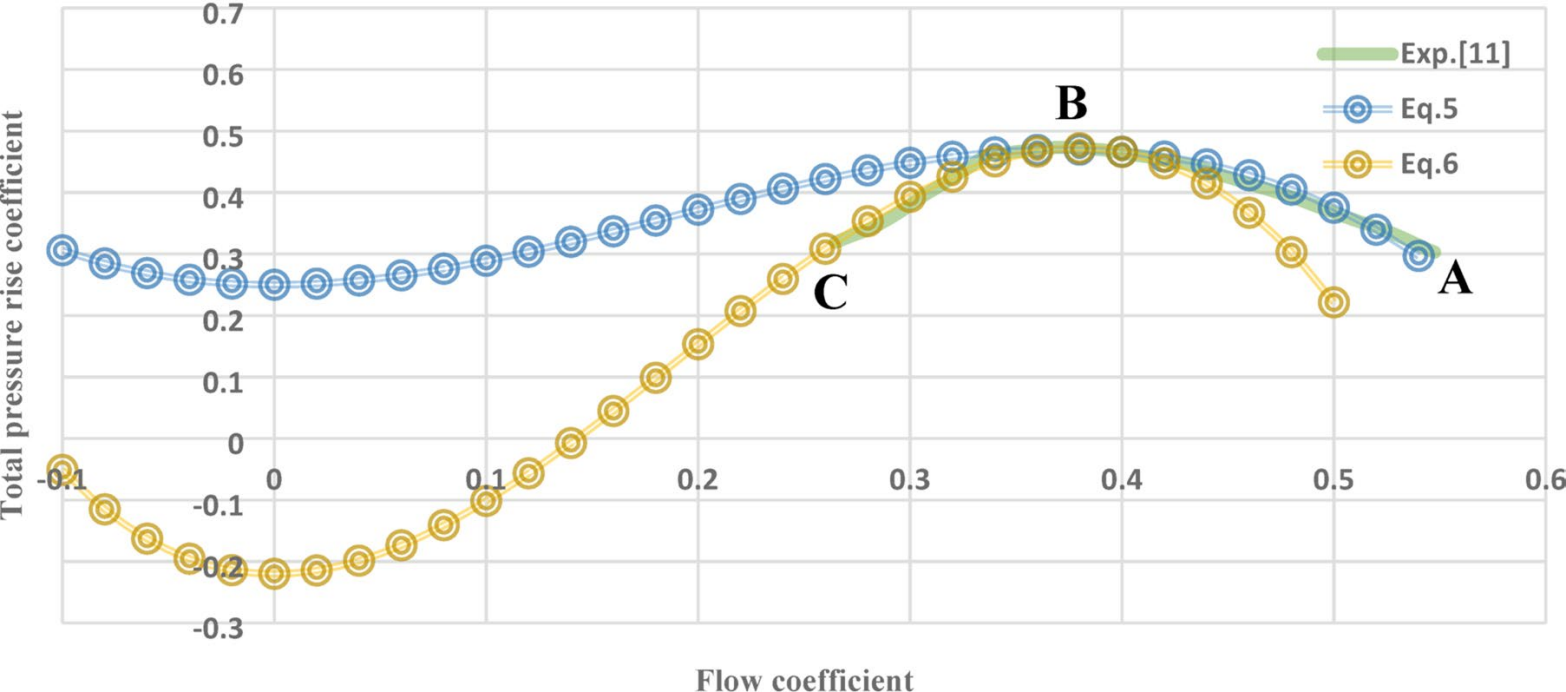
Performance curve approximation.

These curves are sketched in Fig. 7 and the experimental results of Inoue^9^ are also superimposed in this figure. AB and BC parts of the curves correspond respectively to Eqs. 5 and 6 coincide with the experimental curve. The stable part approximation is in close agreement with the experimental data of stable operation (part AB). However, this approximation is disregarded at the unstable part of the curve (positive slope), since the pressures are significantly higher than those of experimental data.

**Fig. 8:**
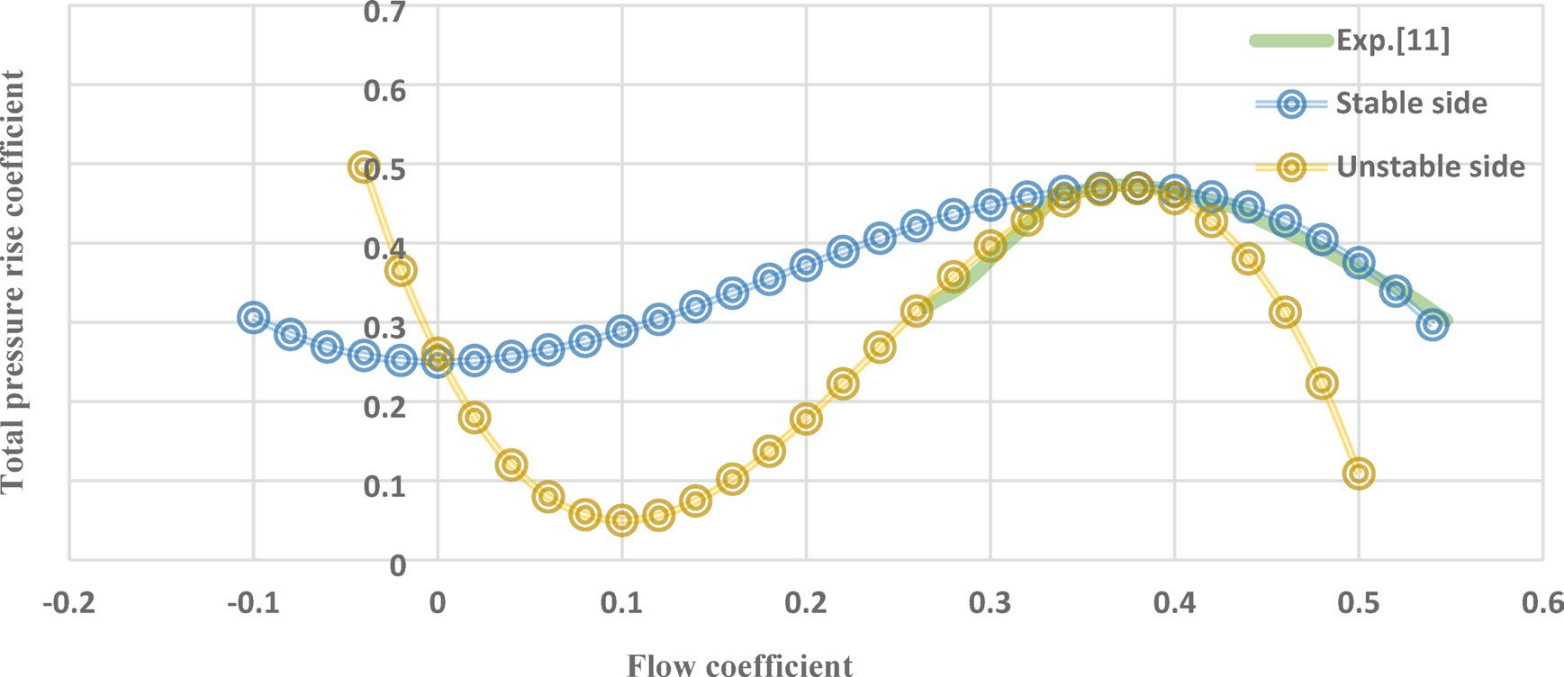
Performance curve approximation with required shift for modelling of unstable operation.

**Fig. 9:**
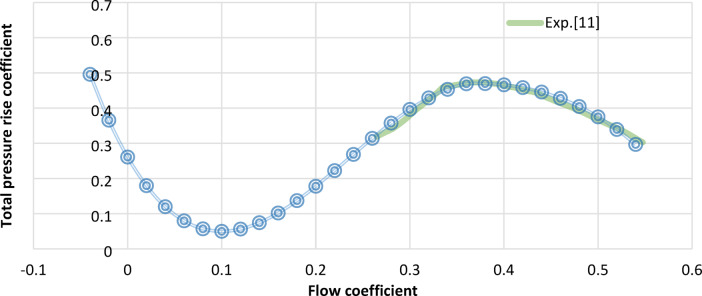
Performance curve approximation used in present method.

Although the left-sided approximation, obtained through plotting Eq. 6, has pressure values close to the experimental signals (part BC), its deviations in the stable operating region (negative slop) reduce its accuracy. Since neither of these curves fit all points, a new method is proposed. This method involves using two distinct third-order polynomial curves for the stable and unstable operational points. To physically meaningful fitting of the unstable points, as shown in Fig. 8, a shift of the curve is considered such that the ${\psi}_{c0}$ remains equal to the stable part approximation. To ensure the best agreement with the experimental curve, an optimization procedure was implemented in MATLAB, where the coefficients were iteratively adjusted to minimize the RMS error between the experimental data and the fitted curve. Furthermore, the constant terms of the two cubic functions were aligned such that the curves are continuous and tangent at the transition (upper extremum) point, guaranteeing smooth behavior across the stable–unstable boundary. Subsequently, Eq. 7 is obtained to approximate also the unstable part of the performance curve, which is shown graphically in Fig. 9.

7 $${\psi}_{c}\left(\phi \right)=0.05+0.21\left[1+\frac{3}{2}\left(\frac{(\Phi -0.1)}{0.1363}-1\right)-\frac{1}{2}{\left(\frac{(\Phi -0.1)}{0.1363}-1\right)}^{3}\right]$$

Based on the proposed method, the compressor performance curve is closely approximated to the experimental data points as presented in Fig. 9. By incorporating this method into the governing equations presented earlier, an accurate model can be developed. It is assumed that the potential function Y can be represented as a single harmonic with an unknown amplitude A and phase r, as described by Eq. 8.

8 $$Y=\mathrm{W} \mathrm{A}\left(\upxi \right)\mathrm{sin}(\theta -r\left(\xi \right))$$

The phase angle r(ξ) is introduced to account for the dynamic nature of perturbations within the system, reflecting their transient and rotational behavior more accurately. Next, a new variable of $J={\mathrm{A}}^{2}\left(\upxi \right)$ is defined to represent the squared rotating stall amplitude. By applying the Galerkin method to simplify the governing equations, the system of equations is transformed into the form of the Ordinary Differential Equations (ODE) of the Moore-Greitzer model. The resulting equations are as follows:

9 $$\frac{d\Psi }{d\xi }=\frac{W/H}{4{B}^{2}}\left[\frac{\Phi }{\mathrm{W}}-\frac{{\Phi}_{\mathrm{T}}}{W}\right]\frac{H}{{l}_{c}}$$

10 $$\frac{d\Phi }{d\xi }=\left[1-\frac{{\Psi -\psi }_{c0}}{H}+\frac{3}{2}\left(\frac{\Phi }{\mathrm{W}}-1\right)\left(1-\frac{J}{2}\right)-\frac{1}{2}{\left(\frac{\Phi }{W}-1\right)}^{3}\right]\frac{H}{{l}_{c}}$$

11 $$\frac{dJ}{d\xi }=J\left[1-{\left(\frac{\Phi }{\mathrm{W}}-1\right)}^{2}-\frac{1}{4}J\right]\frac{3aH}{(1+ma)W}$$

This new system of equations encompasses both types of aerodynamic instabilities (i.e., rotating stall and surge). By incorporating appropriate assumptions, each type of instability can be identified and analyzed via solving these equations.

**Fig. 10:**
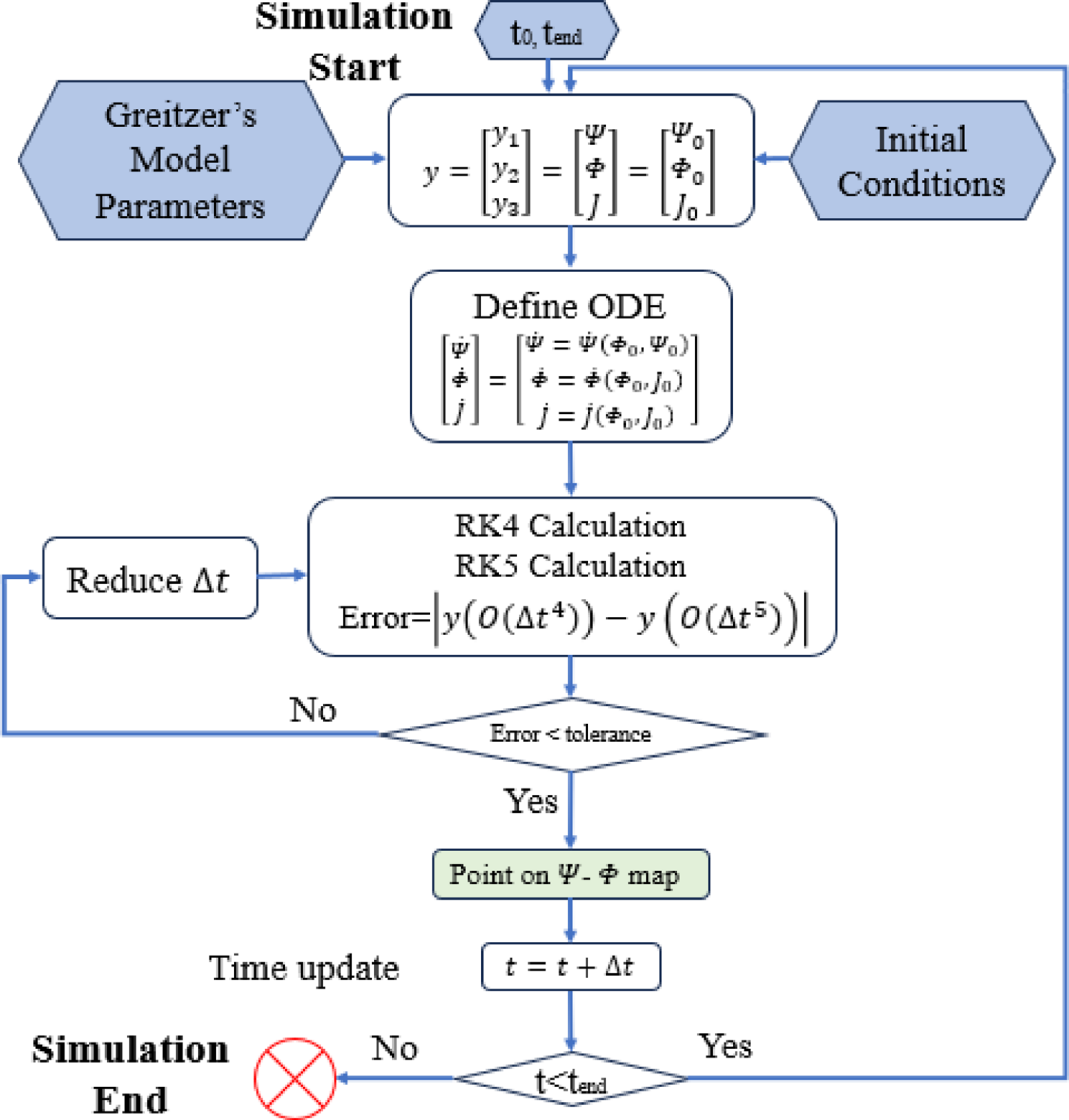
Flowchart of analytical calculations.

This section focuses on development of computational algorithms to solve the governing equations and investigate their implications in scenarios involving rotating stall and surge. The primary objective is to analyze the temporal variations of three key parameters of $\uppsi ,\phi , \mathrm{a}\mathrm{n}\mathrm{d} J$. Figure 10 shows the flowchart of analytical calculations procedure used to develop the computational code, enabling a systematic exploration of the system dynamic behavior.

The simulation starts by defining the required initial values of the variables $\uppsi ,\phi , \mathrm{a}\mathrm{n}\mathrm{d} J$ and the parameters of the Greitzer’s compressor model. The governing equations, introduced by Eqs. 9–11, are represented in the general form of $dy=f(y,t)$, where y is the vector of variables.

12 $$y=\left[\begin{array}{c}{y}_{1}\\ {y}_{2}\\ {y}_{3}\end{array}\right]=\left[\begin{array}{c}\Psi \\ \Phi \\ J\end{array}\right]$$

13 $$\left[\begin{array}{c}{dy}_{1}/dt\\ {dy}_{2}/dt\\ {dy}_{3}/dt\end{array}\right]=\left[\begin{array}{c}\frac{1}{4{l}_{c}{B}^{2}}\left[\Phi -{\Phi}_{T}\right]\\ \left[1-\frac{\Psi -{\Psi}_{c0}}{H}+\frac{3}{2}\left(\frac{\Phi }{W}-1\right)\left(1-\frac{J}{2}\right)-\frac{1}{2}{\left(\frac{\Phi }{W}-1\right)}^{3}\right]H/{l}_{c} \\ J\left[1-{\left(\frac{\Phi }{W}-1\right)}^{2}-\frac{J}{4}\right]\frac{3aH}{\left(1+ma\right)W}\end{array}\right]$$

These equations capture the dynamics of the system and are solved numerically over small-time intervals. The Runge–Kutta (RK4 and RK5) methods are applied to approximate the solution of the ODEs at each time step. If the error between the fourth-order (RK4) and fifth-order (RK5) solutions exceeds the specified tolerance, the time step Δt is reduced to improve the accuracy.

The simulation is conducted over a defined time interval, starting from the initial time t_0_ and progressing to the final time t_end_. Within this interval, the values of $\uppsi ,\phi , \mathrm{a}\mathrm{n}\mathrm{d} J$ are computed at each intermediate time step. These calculated values enable the generation of data points that can be plotted on the Ψ—Φ plane. This graphical representation provides valuable insights into the compressor dynamic behavior of the compressor, enabling the detection and analysis of the potential aerodynamic instabilities, such as rotating stall and surge.

Since one of the input parameters into the Moore-Greitzer model is the steady-state compressor characteristic, the critical *B* value will be unique to a compressor. The purpose of this study is to investigate different values of B and determine the critical value of B for rotor blades row in a low-speed axial compressor test-rig of IUST-ATLab. By determining the B value, the Moore-Greitzer model could be applied for reliable surge and stall protection.

## Validation

**Fig. 11:**
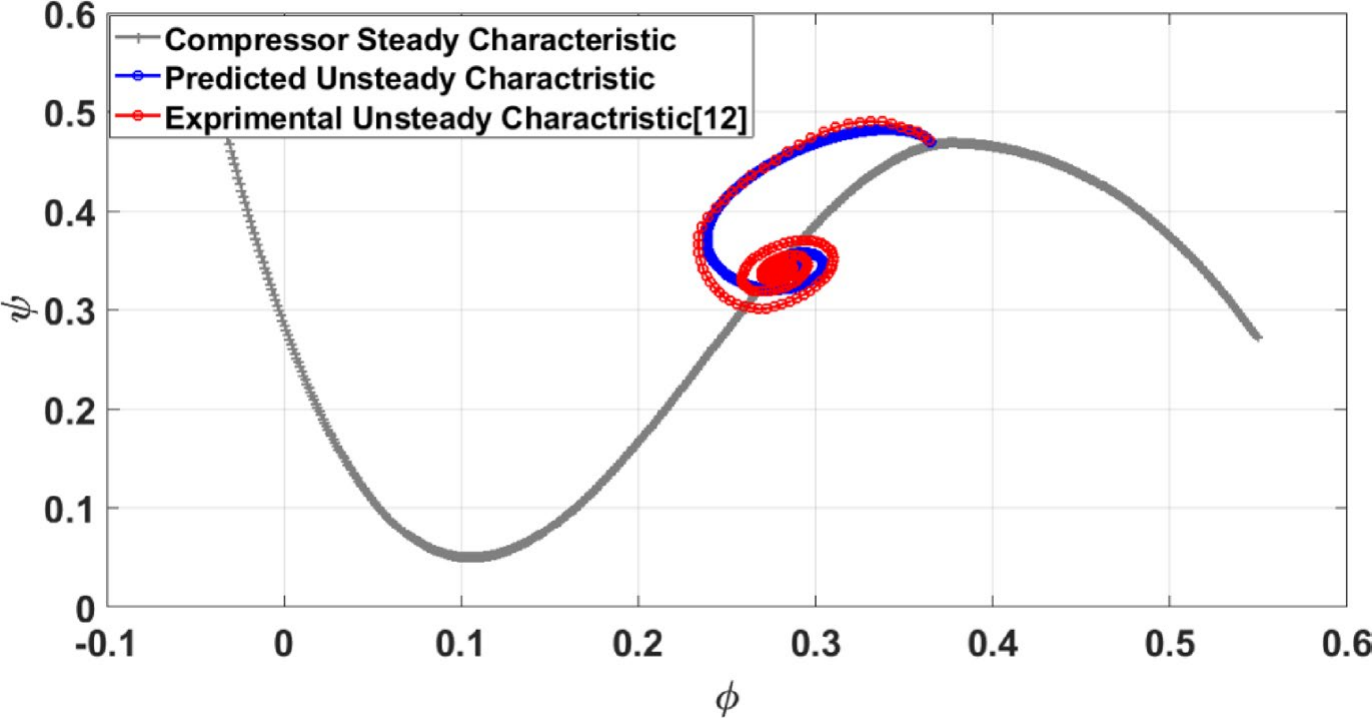
Experimental and predicted unsteady characteristic.

In this section, the proposed compressor modeling approach based on the Moore-Greitzer model, is validated by comparing the analytical simulation results with the experimental findings reported by Taghavi et al.^10^, ensuring the accuracy and reliability of the present model. Specifically, Fig. 11 illustrates this comparison while operating the compressor at a speed of 1300 rpm, in terms of variations of the total pressure rise coefficient (ψ) with flow coefficient (φ). The close alignment between the predicted and experimental results demonstrates the capability of the proposed model for accurate capturing of the compressor dynamic behavior.

**Fig. 12:**
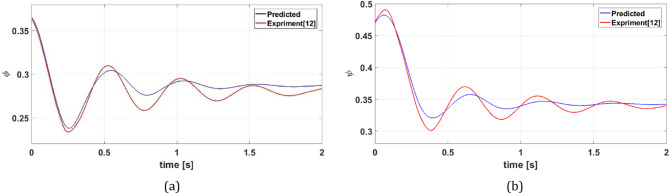
Experimental and predicted system transient.

Figure 12a and b demonstrate the system transient behavior corresponding to B = 0.71. The theoretical prediction, based on the proposed modeling, predicts that at this specific value of B, the compression system will reach an instability at the stall line. Subsequently, transitions to a new stable operating point with considerably lower pressure rise and flow rate. This behavior can be seen in Fig. 12a and b, where the initial drop in the experimental data aligns closely with the theoretical predictions. Upon experiencing instability at the initial operating point, the system undergoes a substantial transient response with eventual settling into a new operating point. At this new point, the system exhibits a damped oscillatory pattern, indicative of a stable equilibrium state.

The RMS error is found to be approximately 0.89% for $\uppsi$ and 0.81% for $\phi$, confirming the predictive accuracy of the modified Moore-Greitzer model in simulating unsteady compressor characteristics and supports its applicability for analyzing and modeling of compressor instability dynamics.

## Complementary results and discussions

In this study the Moore-Greitzer model is modified by introducing two separate third-order characteristic curves for the stable (negative-slope) and unstable (positive-slope) regions of the compressor performance curve, instead of a single cubic polynomial used in the original model. This modification enables a more accurate reconstruction of the compressor characteristic curve. The accuracy of the improved model is evaluated and discussed in the Validation section.

This section presents the procedure for determining the critical value of B parameter and its influence on the dynamic response of the compressor, including oscillation frequency and amplitude. To investigate the limitations of the classical approach, the dynamic responses predicted by the original Moore-Greitzer model (based on Eq. 5) are compared with experimental results, providing a quantitative assessment of the errors arising from the single-cubic approximation. The analysis emphasizes that accurate prediction of the critical value for B and the associated dynamic characteristics is strongly dependent on the fidelity of the performance curve representation, supporting the proposed piecewise, dual-cubic approach.

**Fig. 13:**
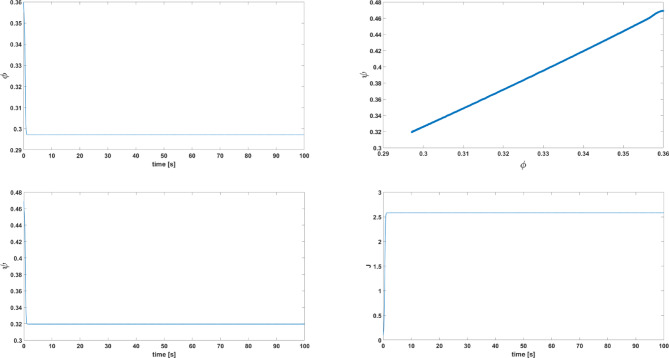
Variations of Ψ, Φ and J for B = 0.1

**Fig. 14:**
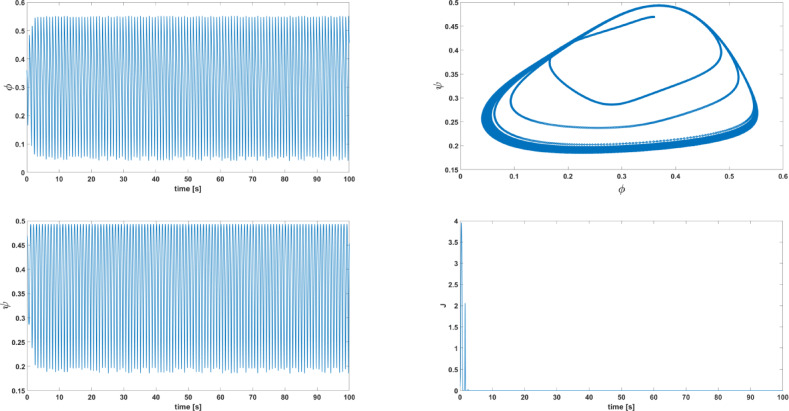
Variations of Ψ, Φ and J for B = 1.1

**Fig. 15:**
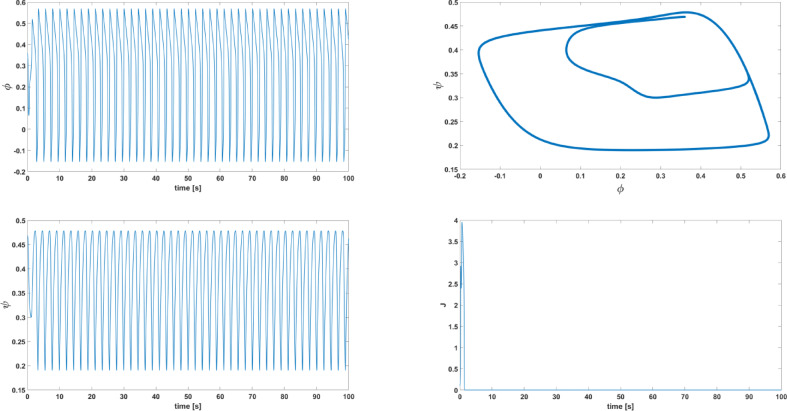
Variations of Ψ, Φ and J for B = 2.1

The critical value of Greitzer’s parameter (B) is determined for both the original and modified models as a critical control factor for determining the dynamic behavior of the low-speed axial compressor. Along with a fixed geometry of the compressor rotor and test rig components, the B value was systematically varied from 0.1 to 2.1 while keeping all other parameters constant in modified model. This range was chosen to identify the critical B-value at which the surge phenomenon initiates. The dynamic behavior and variations in Ψ and Φ are presented in Figs. 13, 14 and 15.

As illustrated in Fig. 13, at low values of B, all quantities quickly stabilize and exhibit a constant trend shortly after the calculations commence. By incrementally increasing the B value, the dynamic behavior of the aforementioned parameters is monitored to determine the critical range for the B parameter. These latter results are presented in Figs. 14 and 15.

**Fig. 16:**
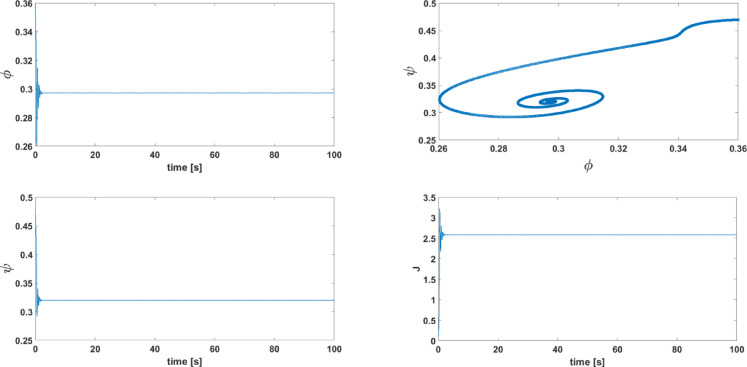
Variations of Ψ, Φ and J for B = 0.6

**Fig. 17:**
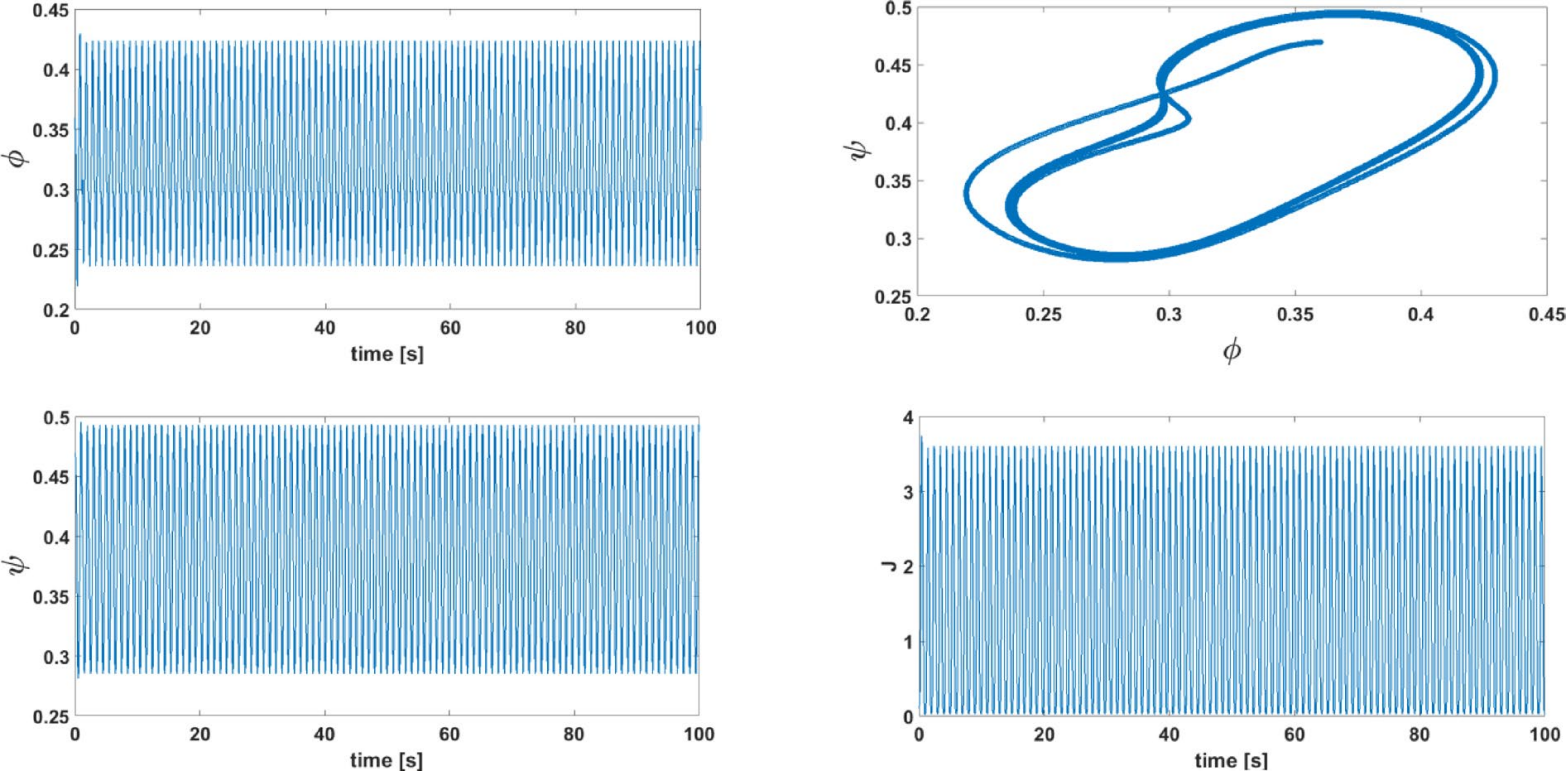
Variations of Ψ, Φ and J for B = 0.8

The analysis reveals that at higher values of B, the compression system no longer achieves a stable operating point. Instead, the system exhibits limit cycle oscillations which characterizes the commencement of the surge. Results demonstrate that as the stall line is approached, the system instability intensifies with a rapid and abrupt transition to surge cycle oscillations. As B increases further, the frequency and shape of the limit cycles will also change. For a more detailed investigation of the system behavior, it is more advantageous to focus on smaller increments of B within the range of 0.2 to 1. This range is particularly critical, as significant changes in the compressor operational dynamics occur within this range. Exploring this interval with finer resolution provides deeper insights into the transition mechanisms and instability thresholds. These results are presented in Figs. 16 and 17. At B = 0.6, the Φ-t and Ψ-t graphs exhibit behavior that stabilizes towards a limit, a trend similarly observed in Ψ-Φ graph. The flow disturbances remain in a uniform state with a non-zero constant value, representing the disturbance range characteristic of the rotating stall condition in the compressor.

As the B value increases to 0.8 the squared amplitude of the flow disturbances transitions to an oscillatory state (see Fig. 17). Comparing Figs. 17 and 14 reveals that as B increases, the range of J disturbances decreases, initiating a damping process. This process continues until B = 1.1 where the disturbance range diminishes entirely, as depicted in Fig. 14.

The analyses further show that these disturbances fluctuate within the same frequency spectrum as variations in the Φ and Ψ, highlighting the presence of a classic surge, a condition in which both the rotating stall and surge instabilities coexist. Beyond the onset of classic surge, the initial fluctuations evolve into a damping state, where the disturbance amplitude approaches zero.

**Fig. 18:**
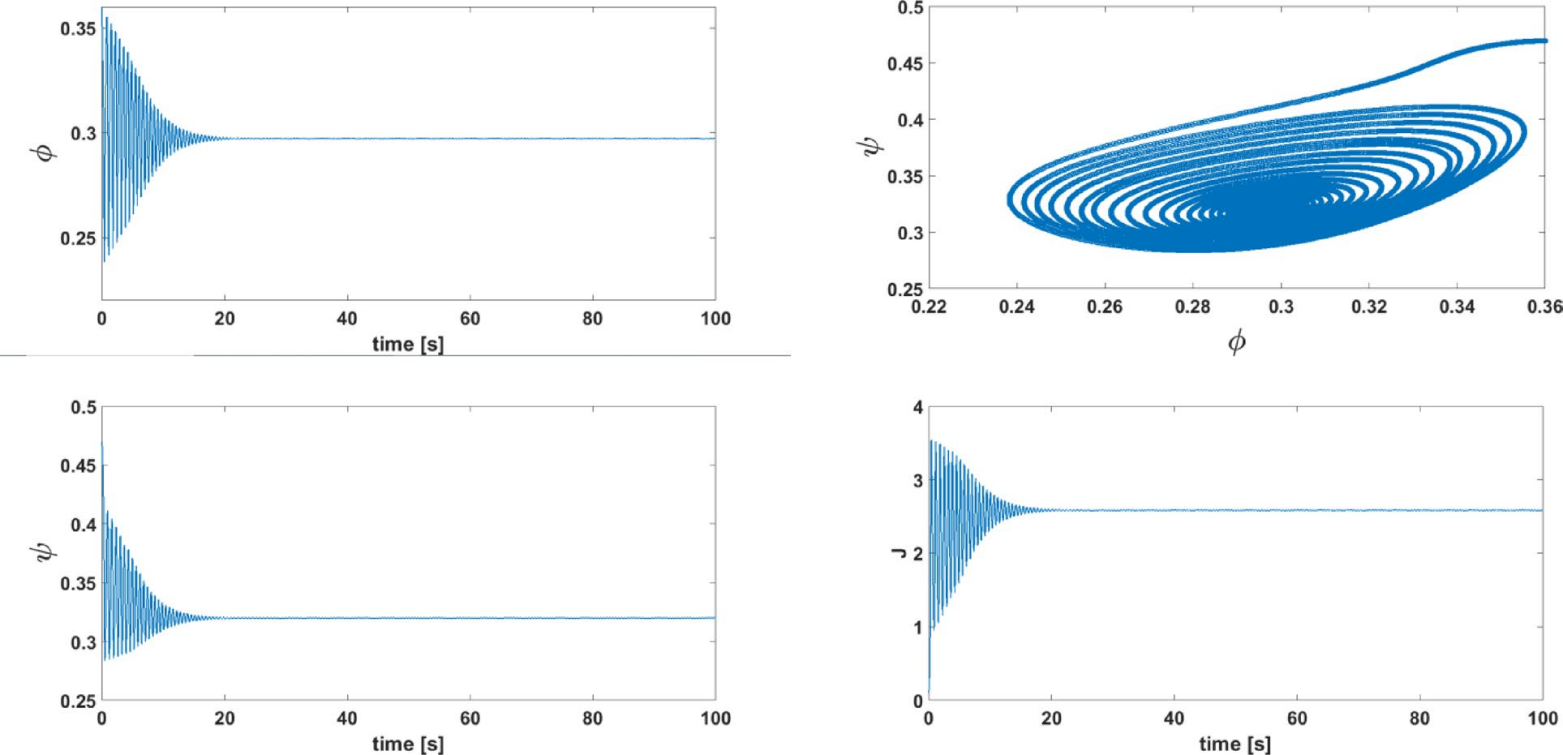
Variations of Ψ, Φ and J for B = 0.71.

**Fig. 19:**
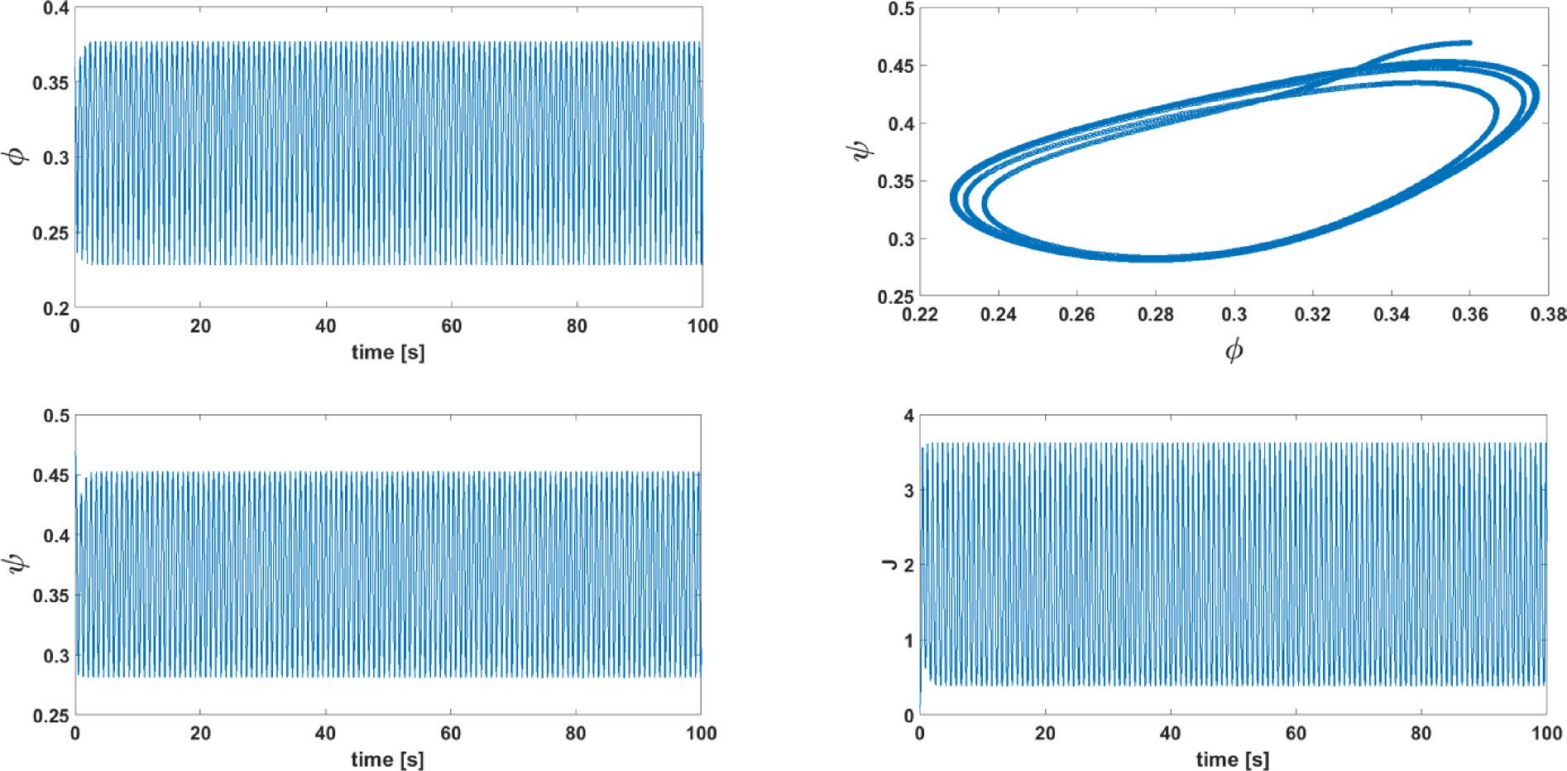
Variations of Ψ, Φ and J for B = 0.72.

To identify the precise critical value of B, additional simulations were performed with B values ranging from 0.6 to 0.8 in 0.01 increments. For the sake of brevity, only the graphs corresponding to values near the critical point are presented in Figs. 18 and 19. These results provide further clarity on the transition from rotating stall to surge. A detailed analysis of the figures highlights several key observations regarding the stability behavior of the compression system. Firstly, as the parameter B increases, the amplitude of fluctuations in the flow coefficient (Φ) and pressure coefficient (Ψ) also grows. For B values below a critical threshold, denoted by B_critical, these fluctuations exhibit a damped response, indicating that despite the initial disturbances in Φ and Ψ, the system eventually stabilizes and reaches a steady operating limit.

Furthermore, an examination of the characteristic curves for different B values reveals that the disturbance loops in the Φ-Ψ plane become increasingly pronounced with higher B values. Both the amplitude and the number of cycles in these loops expand, indicating a progression toward the onset of the surge cycle. This evolution is accompanied by a rising frequency of oscillations, with disturbances transitioning from a damped state to a sustained oscillatory state, as depicted in Fig. 19. This behavior indicates that the compression system approaches the surge cycle, where oscillations persist without damping. Based on these observations, a critical threshold of B = 0.71 is identified for the modeled compressor. For B ≤ 0.71, the system exhibits rotating stall, while for B > 0.71, the system transitions into the surge. At higher B values, the severity of the surge cycle increases, with larger oscillation amplitudes and sustained instability.

**Fig. 20:**
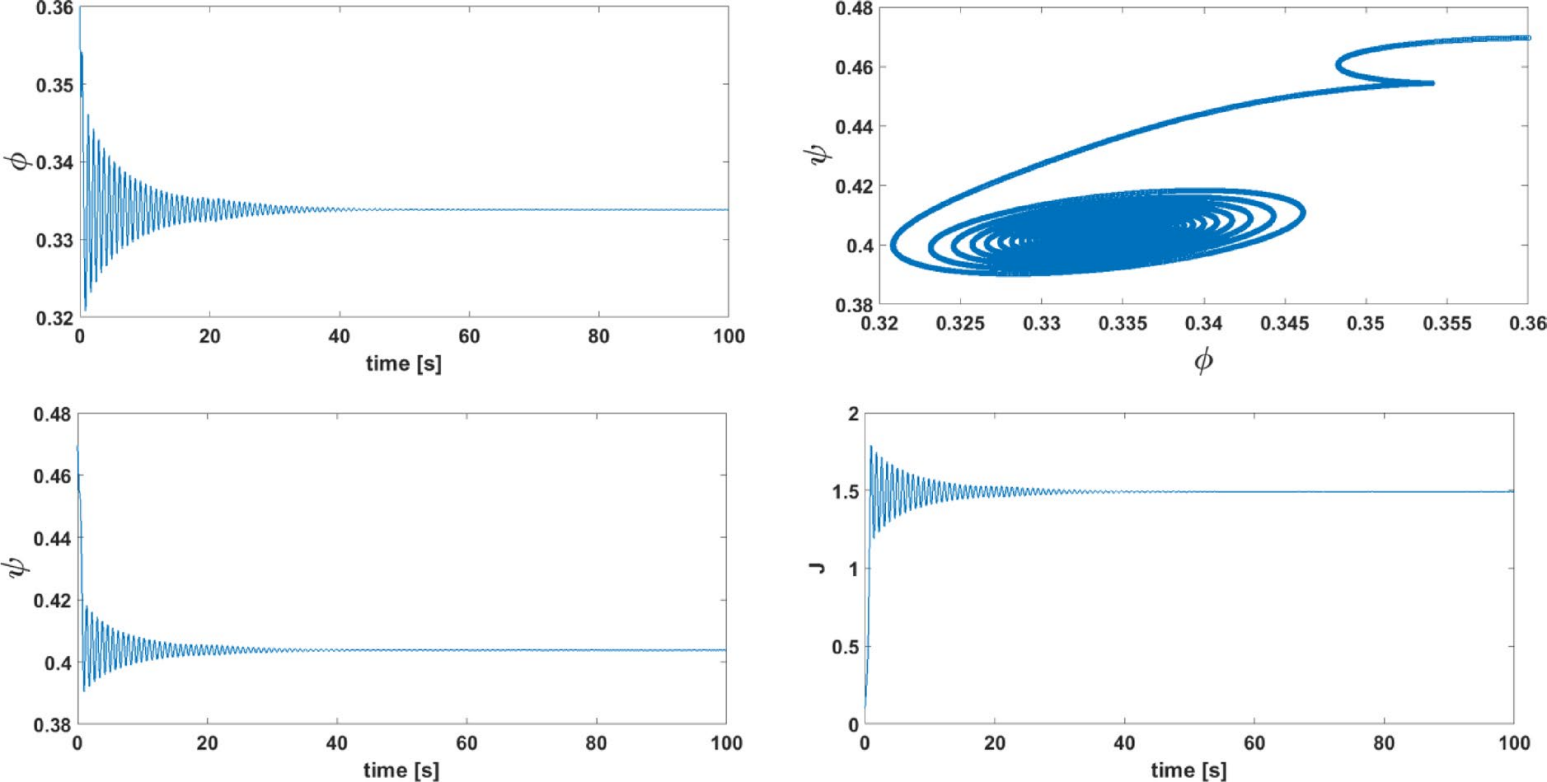
Variations of Ψ, Φ and J for B = 0.78-original model (Eq. 5).

**Fig. 21:**
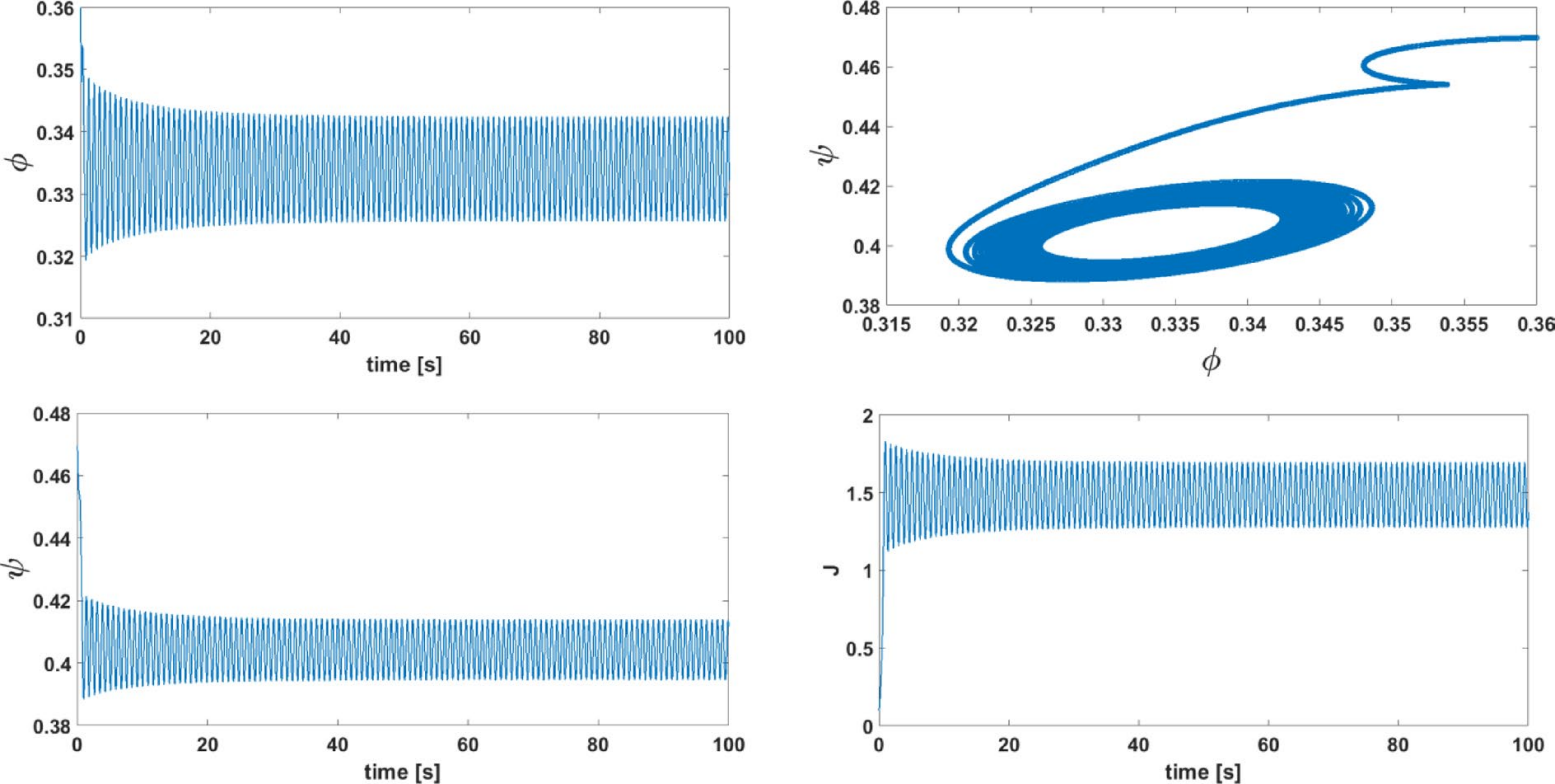
Variations of Ψ, Φ and J for B = 0.79-original model (Eq. 5).

The same procedure was repeated using the original Moore-Greitzer model, considering the Eq. 5 as the compressor characteristic curve. The critical value of B was determined from this analysis equal to B = 0.78 as illustrated in Figs. 20 and 21.

**Fig. 22:**
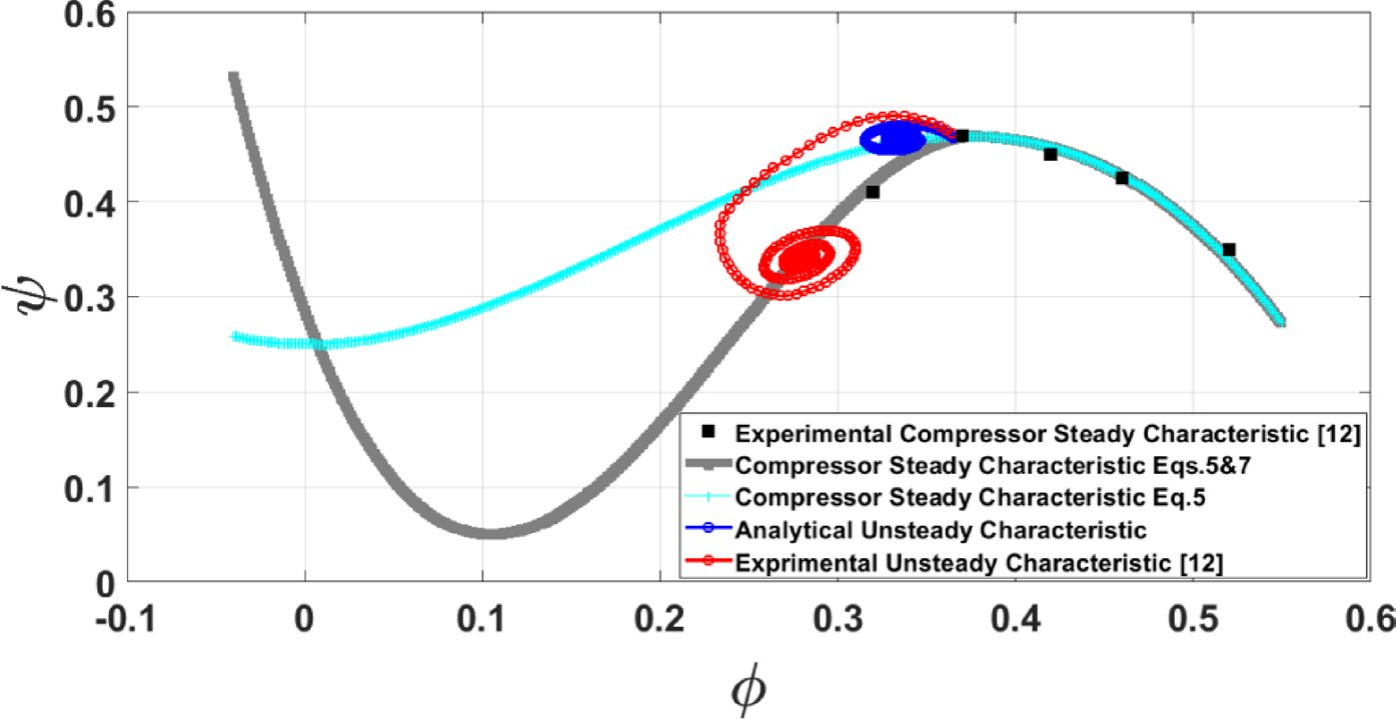
Experimental and analytical unsteady characteristic.

**Fig. 23:**
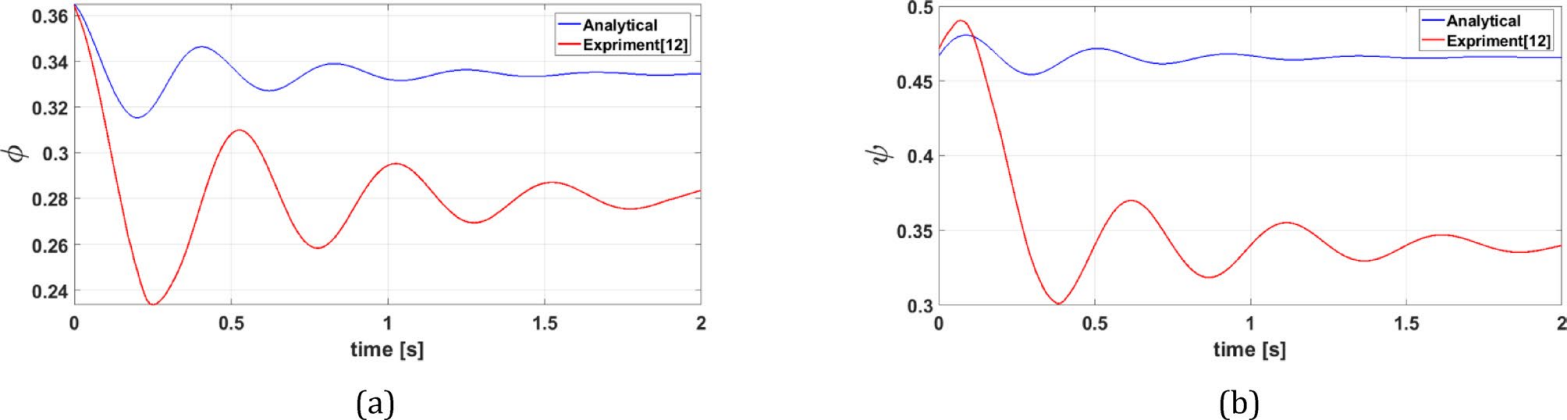
Experimental and analytical system transient.

The comparison of the analytical simulation results based on original model with the experimental findings reported by Taghavi et al.^10^, are presented in Figs. 22 and 23. The predicted dynamic behavior of the compressor shows RMS errors of approximately 11.97% for $\uppsi$ and 5.65% for $\phi$.

The results obtained through using a single third-order curve demonstrate the limitations of original model in accurate prediction. In contrast, as presented in validation section, the proposed dual-cubic approach yields a prediction within less than 1% deviation from the experimental data. Therefore, while both models follow the same fundamental Moore-Greitzer model, the proposed modification enhances the fidelity of the characteristic curve representation and thus allows for more precise determination of B parameter and the compressor dynamic behavior under unstable flow conditions.

These results provide valuable insights into the dynamic behavior of the compression system, showcasing the practical applicability of the refined model in real-world scenarios.

## Conclusion

In this study, a modification to the Moore-Greitzer Model is presented, introducing a refined mathematical framework for dynamic modeling of flow instabilities in low-speed isolated axial compressor rotor blades rows. This approach addresses the key limitations of the Moore-Greitzer Model and offers modifications to this method through utilizing experimental performance curves together with instantaneous velocity and pressure signals. The main conclusions drawn from the present research are listed below.

- The proposed modification enables the precise determination of the B-Greitzer parameter and allows for accurate dynamic modeling of both the rotating stall and surge instabilities.
- Analytical simulations highlight the importance of B value equal to 0.71 as a critical boundary which marks transition between rotating stall and surge modes.
- The analysis reveals that the B parameter influences both oscillation frequency and amplitude.
- By increasing B, the amplitude initially rises sharply to a peak value and subsequently exhibits a slight decline. A similar trend is observed in the oscillation frequency.
- Using this refined modeling approach, the RMS error is found to be approximately 0.89% for $\uppsi$ and 0.81% for $\phi$, confirming the accuracy of the method.

In the compressor studied here, the loading coefficient lies within a practically relevant range (0.36–0.47), for which modal-type instability is commonly observed as the dominant large-scale mechanism. Modeling of local and highly nonlinear phenomena such as spike-type stall is beyond the scope of the present lumped-parameter framework. The proposed refined model provides a practical and reliable framework that can be employed by researchers for prediction of surge and modal-type stall instabilities and designing advanced control systems, enabling compressors to operate more safely and efficiently near their instability limits. This has direct implications for enhancing the performance, efficiency and reliability of turbomachinery in industrial applications.

## Data Availability

The associated data and analysis code generated for current study are publicly accessible in the Zenodo repository with the 10.5281/zenodo.19605877
